# Hydroxytyrosol: Health Benefits and Use as Functional Ingredient in Meat

**DOI:** 10.3390/medicines5010013

**Published:** 2018-01-23

**Authors:** Lorena Martínez, Gaspar Ros, Gema Nieto

**Affiliations:** Department of Food Technology, Nutrition and Food Science, Veterinary Faculty University of Murcia, Regional Campus of International Excellence “Campus Mare Nostrum” (Economy based on agri-food), Campus de Espinardo, 30100 Espinardo, Murcia, Spain; lorena.martinez23@um.es (L.M.); gros@um.es (G.R.)

**Keywords:** hydroxytyrosol, antioxidant, antimicrobial, meat, preservative, health

## Abstract

Hydroxytyrosol (HXT) is a phenolic compound drawn from the olive tree and its leaves as a by-product obtained from the manufacturing of olive oil. It is considered the most powerful antioxidant compound after gallic acid and one of the most powerful antioxidant compounds between phenolic compounds from olive tree followed by oleuropein, caffeic and tyrosol. Due to its molecular structure, its regular consumption has several beneficial effects such as antioxidant, anti-inflammatory, anticancer, and as a protector of skin and eyes, etc. For these reasons, the use of HXT extract is a good strategy for use in meat products to replace synthetics additives. However, this extract has a strong odour and flavour, so it is necessary to previously treat this compound in order to not alter the organoleptic quality of the meat product when is added as ingredient. The present review exposes the health benefits provided by HXT consumption and the latest research about its use on meat. In addition, new trends about the application of HXT in the list of ingredients of healthier meat products will be discussed.

## 1. Introduction

Meat and meat product consumption provides high-quality proteins (20–25%), minerals (Fe- heme, Mg, K, Zn and Se) and vitamins (A, thiamine, riboflavin, niacin, retinol, B6, folic acid, B12, D and K) necessary for a balanced diet. However, these products usually are rich in saturated fatty acids, and recently, the International Agency for Research on Cancer (IARC) under the World Health Organization (WHO) has classified processed meat as a carcinogen (Group I) and red meat as possible carcinogen (Group 2A) (October 2015) [1]. In fact, carcinogenic compounds in meat could be added during their processing (synthetic additives), but they also can be formed during their storage through lipid and protein oxidation, or during cooking through the Maillard reaction [2,3]. In this way, synthetic additives such as sulphites, BHT (butylated hydroxytoluene) and BHA (butylated hydroxyanisole) are added in meat product formulation to preserve them. The use of these synthetic additives has given rise to social concern by consumers, due to studies that correlates their consumption with disease development (asthma, hyperactivity, cancer, etc.) [4,5,6]. On the other hand, lipid peroxidation in meat and meat products happens through the radical chain reaction mechanism, although oxygen presence accelerates this process. This oxidation is due to several factors such as polyunsaturated fatty acids concentration (PUFA), the deficit of antioxidants in animal feed (tocopherol, rosmarinic acid) and a high concentration of prooxidants, free radicals or added salt (NaCl). At the same time, these reactions produce reactive oxygen species (ROS) like hydroxyl radical, superoxide anion, ferryl and perferryl species, lipid peroxyl radical and secondary products like reactive carbonyl species (MDA (malondialdehyde) and 4-HNE (4-hydroxynonenal)) responsible for the rancid flavour in aged meat.

Although protein oxidation has received less attention, it has a huge influence on quality of meat [7]. Protein oxidation has been defined as a covalent modification of protein induced either directly by reactive species or secondary products of oxidative stress. The same oxidants that induce the lipid peroxidation produce this alteration, and carbonyl formation is a common reaction in protein oxidation. Furthermore, proteins can react with secondary products of lipid peroxidation like aldehydes and ketones to produce complexes between proteins, proteins and carbonyls or proteins and lipids. In muscle fibres, hydroxyl radical (OH) in presence of Fe or Cu or ROS causes modifications of amino acids, like methionine, lysine, arginine, histidine, tryptophan, valine, serine and proline. This reaction increases proteolytic enzymes and protein polymerization, which produces soluble aggregates, that promotes gelation and emulsification that modifies the texture and toughens the meat [8,9,10]. But this not only is critical for organoleptic quality, but it might have an impact on human health and safety. For example, during cooking it increases free radical generation while it decreases the antioxidant compounds in meat, which contribute to protein oxidation.

Therefore, natural antioxidants can prevent lipid peroxidation on different ways: preventing chain inhibition by scavenging initiating radicals, breaking chain reaction, decomposing peroxides, decreasing localized oxygen concentrations and binding chain initiating catalyst such as metal ions. Therefore, the use of natural preservatives to keep the shelf life of meat has exhibited similar antioxidant properties compared to some synthetic additives. For this reason, it is a promising tool due to many fruits (grapes, grape seed, pomegranate, date, kinnow mandarin), vegetables (broccoli, potato, drumstick, pumpkin), herbs (olive leaf, acerola, grape seed, cocoa, green coffee, *Ginkgo biloba*, etc.) and spices (rosemary, green tea, black pepper, garlic, oregano, cinnamon, sage, thyme, mint, ginger, clove) reported antioxidant properties in meat products [11,12,13,14].

**Figure 1:**
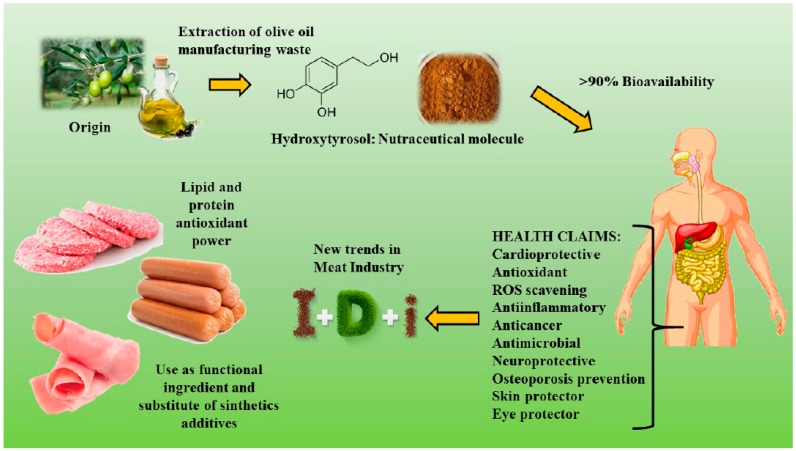
Graphical abstract of the use and health benefits of Hydroxytyrosol to prevent lipid and protein oxidation and substitution of synthetic additives in meat products.

**Figure 2:**
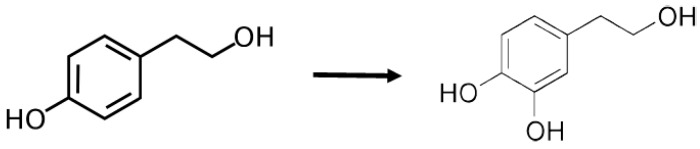
Chemical structures of TYR and HXT: phenolic compound from olive leave and olive oil. TYR: tyrosol (left); HXT: hydroxytyrosol (right).

One of most potent natural antioxidant extracts is hydroxytyrosol (or 4-(2-dihydroxyphenyl)ethanol) (HXT)), just below gallic acid (Figure 1). This compound is ten times more antioxidant than green tea and two times more than coenzyme Q10 [15], additionally HXT scavenging ability is comparable to oleorupein and catechol. HXT is a phenylethanoid with demonstrated antioxidant properties in vitro, it is found in olive leaf and oil from this fruit, responsible for intense flavour and aroma, being oleuropein precursor [16,17]. In addition, it has demonstrated this capacity in vivo in several studies in rats, such as Merra et al. or Lemonakis et al. [18,19], that showed the power of HXT to reduce the risk to suffer metabolic syndrome. In its chemical structure, this compound has an additional OH group in its benzene ring, compared to the tyrosol (TYR) (Figure 2). Therefore, it obtains a greater function as a free radical scavenging, increasing its antioxidant power, as well as its efficacy under stress conditions.

In this way, this extract has previously demonstrated its antioxidant capacity in meat products rich in unsaturated fatty acids like sausages and frankfurters with added HXT, nuts and extra virgin olive oil [20,21]. Moreover, HXT is an antioxidant compound linked to certain minerals, such as gluconate Fe (II) in black olives, which catalyzes the oxidation of this compound, so it is possible that HXT influence on biological bioavailability of some minerals and trace elements [17].

The objective of this paper is to review the latest literature about HXT consumption benefits, its extraction from olive leave and other sources and its used as natural antioxidant in meat and meat products as substitute of synthetics additives, with emphasis on new trends and future perspectives in investigation and meat industry.

## 2. The Role of HXT in Diet

Since many years ago it is known that several aspects about the Mediterranean diet have been associated with a minor risk of cardiovascular diseases and cancer (colorectal, breast, prostate or pancreas, between others) [22] (Figure 1). In particular, olive oil as principal component of this dietary model plays a key role in these benefits, due to its composition of fat, richness in monounsaturated fatty acids (oleic acid) and another micronutrient from non-saponifiable fraction such as squalene, phytosterols, tocopherols and secoiridoids.

HXT is a secoiridoid and a potent antioxidant that acts as main component from phenolic fraction of extra virgin olive oil (EVOO). This compound is derivate from hydrolysis of oleuropein and its concentration in EVOO and table olives can change according to several factors: altitude and latitude of olive tree harvest, the variety of olive, the collection time and the processing conditions [23,24]. For example, the HXT concentration in EVOO is 14.32 ± 3.01 mg kg^−1^, while in refined virgin oil is 1.74 ± 0.84 mg kg^−1^ [25]. In the same way, the concentration in Greek black olives is 100–340 mg kg^−1^, in Spanish green olives is 170–510 mg kg^−1^ and 250–760 mg kg^−1^ in Greek kalamata olives [26]. Taking into account that the average consumption of EVOO and olives in Spain are 15 g/day^−1^ and 7 g/day, respectively [27], it can be estimated that the HXT consumption per day is 5.6 mg (0.3 mg from EVOO and 5.3 mg from olives), approximately.

In addition, HXT has a bioavailability of 99%, so this compound is easily integrated by human body [28]. In the same way, it was corroborated by Khymenets et al. [29] in their study about human HXT absorption and excretion from a nutraceutical, that HXT is a phenolic compound resistant to gastric juices and to passing through the digestive system, consequently it is bioavailable and is recovered in the urine chiefly as 3’-sulphate. Therefore, it can be confirmed that the consumption of 5.6 mg day^−1^ of HXT can bring great benefits on an organism’s functioning.

## 3. The Role of HXT in Health

HXT has a great bioactive power due to its great antioxidant capacity due to its protective function in cells, its structural affinity to some compounds (for example, with dopamine, it replaces its amine group (NH_2_) by hydroxyl group (OH)), it has a simple molecular structure and it is present in organism (e.g., iris of the eye) so it is easy to assimilate by the human body, reaching blood plasma in 15 or 20 min until its elimination 6–8 h later by the renal or digestive system, so it does not present accumulation or toxicity problems. In addition, HXT is an amphipathic, water-soluble and fat-soluble molecule, because of it has a lipophilic end and another hydrophilic end, which makes it a good transporter of substances through the human body, therefore, it can penetrate the cellular membrane easier. These structural and molecular features of HXT make its consumption provide many beneficial effects in the organism [30].

Firstly, in 2012, EFSA (European Food & Safety Authority) accepted that HXT acts as protector of the cardiovascular system, avoiding oxidation of LDL cholesterol by free radicals, maintaining normal blood HDL cholesterol concentrations and preventing atherosclerosis [31]. In addition, HXT consumption can regulate glutathione concentration and provides antioxidant enzymes to adipose tissue [32]. Moreover, in other in vivo study with experimentally induced diabetes mellitus in rats, it was shown that the HXT consumption influenced the major biochemical processes leading to diabetic vasculopathy and reduced cell proliferation in the vascular wall [33]. It is confirmed the prevention against damage by oxidative stress induced by this compound, which acted as regulator of cell protection and damage induction, controls the intracellular redox state. This could influence the prevention of diseases such as cancer, diabetes, inflammation or cardiovascular and neurodegenerative diseases, which aetiology and progression has been linked to the production of ROS on damaged tissues. So, the regular consumption of HXT can help to avoid cardiovascular diseases and diabetes mellitus [34].

Secondly, HXT has the capacity to inhibit COX (cyclooxygenase) and LOX (lipoxygenase) enzymes of arachidonic acid (AA), reducing the oxidative deterioration characteristic of inflammations [35]. At the same time, HXT stimulates the production of chondrocytes by regeneration and repairing the articular cartilage. During physical exercise, HXT helps to increase the production of glutathione and to reduce the production of lactic acid and the consequent muscular atrophy [36].

Because of the connection between cellular oxidation, inflammation and the formation and development of a tumour, it can have a clear idea of the anticancer ability of this extract. Moreover, HXT alters a tumour’s eicosanoid biosynthesis and shows an inhibition of tumour cell proliferation [37]. At the same way, the daily consumption of 50 µM–100 µM of HXT, through the intake of EVOO and table olives, has showed antiproliferative activity, apoptotic activity and inhibition of metastasis of leukemia cells (HL60), adenocarcinoma cells (HT29), human colon cancer cells (Caco-2 and HT115) [38,39] and in breast cancer cells (MCF10A, MDA-MB-231 and MCF7) [40].

On the other hand, HXT is protective against neurodegenerative damage and cognitive decline associated with age or diseases like Alzheimer or Parkinson. It is due to the fact that HXT protects brain cells from lipid peroxidation because it is able to cross the blood–brain barrier [41].

Another notable property of HXT is its antimicrobial capacity and acts by inhibiting the growth rate of bacteria in humans, as demonstrated in the research of Medina et al. [42], (against *Clostridium perfringens*, *Escherichia coli*, *Staphylococcus aureus*, *Salmonella enterica*, *Yersinia sp*. and *Shigella sonnei*) and Brenes et al. [43] (against *Helicobacter pylori*). There are also studies with its antifungal properties against *Mycoplasma hominis* and *Pneumoniae fermentans* [44], and against *Fusarium sambucinum*, *Vericillium dahliae* and *Alternaria solani* [45]. Therefore, HXT consumption has an antimicrobial effect that can avoid infections in the respiratory, intestinal and genital systems and it strengthens the immune system. In addition, HXT can act against virus like HIV. Lee-Huang et al. [46] demonstrated that HXT and oleuropein inhibit the entry and integration of virus, as inside as outside cells.

Finally, HXT also helps to prevent osteoporosis because its consumption has positive effects on the formation and growth of bones [47]. In addition, the retina is also protected by HXT and it is beneficial for ocular health specifically in the regeneration of the retinal pigment epithelium, macular degeneration and glaucoma, caused by oxidative stress [48]. Furthermore, HXT has dermoprotective effects: it protects against UV rays, it reduces the pigmentation of the skin, it protects against oedema and erythema caused by excessive sun exposure, it may even be effective in treating psoriasis, it has anti-aging effect and it stimulates the production of cell-survival promoting proteins [49] (Figure 1).

## 4. Preparation of HXT Extract

The highest concentration of HXT is found in olive leaves. However, it is lost during oil manufacturing after the waste of the pomace and vegetable water as residues of EVOO, or refined olive oil production. In order to take advantage of this waste and minimize losses to the oil industry, these residues are used by ingredient companies to obtain natural extracts used by the cosmetic, pharmaceutical and food industries (Figure 1).

HXT can be obtained from vegetation water or from olive leave using different ways. It is based in three phases: first, a phenol-rich liquid is obtained as the raw material for the extraction and purification of phenolic compounds; a second phase enables a HXT enriched extract and a HXT with 3,4-dihydroxyphenylglycol (DOPEG) mixture to be obtained, and HXT acetate is produced. In the third phase, highly pure HXT-DOPEG and HXT acetate are obtained. These phases comprise extraction, reaction, concentration, adsorption and desorption, using mixtures of ion exchange resins, adsorption in nonionic resins and a polymer phenolic fraction, membranes of reverse osmosis and evaporators. This methodology is an example of extraction method for HXT from vegetation waters and *Olea europaea* L. subproducts that it is under intellectual property of the Spanish Research Council (Consejo Superior de Investigaciones Científicas, CSIC) [50].

From the industrial point of view, the extraction method depends on the source used for HXT [21]. The first one obtains HXT from olive waters during fruit processing (separating the oil from wet centrifugation). It is used a solvent extraction and purification process, including, crystallization and clarification steps. For that, the original plant material (vegetation water) is dried and suspended in ethanol. The filtered hydroalcoholic solution is concentrated under vacuum until obtain a syrup with a HXT percentage around 20–25%. Among the characteristic polyphenolic compounds from olive oil that this extract contains are large quantities of fulvic acids. HXT can be also obtained from olive leaves (dehydrated) by hydroalcoholic extraction and subsequent hydrolysis. The hydroalcoholic solution obtained contains as main active compound the oleuropein that is present in the leaves. 

Another form to obtain HXT extract can also be through olive waters (fruit processing) by liquid–liquid extraction with ethanol. For that purpose, the original plant material (vegetation water) is concentrated in vacuum at temperature of 50–60 °C until a syrup of 65–70% °Bx (*w*/*w*) is obtained. This syrup is suspended in ethanol and the supernatant is removed, which is concentrated in vacuum and finally a hygroscopic solid.

## 5. Use of HXT Extract as Functional Ingredient in Meat

Firstly, AECOSAN (Spanish Agency for Consumer Affairs, Food Safety and Nutrition) accepted using of HXT extract as functional ingredient in 2015 [51], while in 2017, EFSA (European Food & Safety Authority) has confirmed that its use does not provided negative effects on health of consumers [52].

Among all of the literature on this topic, it has been selected as the most innovative and current (Table 1). For example, Rounds et al. [53] researched the antimicrobial activity of HXT (10,000 and 30,000 ppm) in ground beef patties inoculated with *E. coli.* They showed that the cell count was reduced 3% more than the control at the same conditions and the amine formation was 50.6% lower than control.

However, the antimicrobial capacity of HXT has already been demonstrated in several matrices, so after this researcher focused on studying the shelf life of meat products in which synthetic additives have been replaced by compounds derived from olive: HXT, tyrosol, oleorupein, verbascoside and pinoresinol. Such as, in the work of Chaves-López et al. [55], they elaborated fermented sausages with HXT (100.23 ppm) and proved that lipid oxidation was lower (12–38%), while volatile compounds were reduced and colour redness was increased. On the other hand, Muíño et al. [56] analysed lamb meat patties enriched with omega 3 (fish oil) and HXT (100, 200 and 400 ppm). They found that antioxidant activity of patties increased with HXT presence and lipid oxidation was lower at day 3, 6 and 9 of storage compared with control samples. In addition, patties with HXT kept the colour and texture stable while the odour and flavour were modified by the extract. Similar results were obtained in pork sausages enriched with HXT (75 and 150 ppm) and stored for 14 days, even though Balzan et al. [57] also showed how this extract had an inhibitory effect on lipolytic activity and microorganism growth. Finally, on chicken frankfurters, Nieto et al. [20,21] proved that a small quantity of HXT (50 ppm) is enough to maintain the colour and reduced the lipid and protein oxidation until 21 days if it is combined with other ingredients like EVOO (extra virgin olive oil) and/or walnuts. At the same way, they compared three extracts: HXT 23% from olive waters, HXT 7% from olive leaves and HXT 7% from olive waters; and they reported that HXT 7% from olive waters was more sensory acceptable than the others.

In addition, meat can be enriched exogenously through the use of natural extracts to replace synthetics additives or endogenously through the animal diet. It is the case of Branciari et al. [58] whose research was based on feeding 297 chickens during 21 days with three treatments: basal diet, diet supplemented with 82.5 g/kg of “paté cake” rich on olive phenols (4.6 ppm HXT), and diet supplemented with 165 g/kg of the same product (9.5 ppm HXT). They showed that chicken feeding with olive phenols had a higher weight; they had a higher antioxidant capacity while lipid oxidation was lower. It is important to remark that the sensory quality of meat was not modified by olive feed.

Taking into account previous studies, HXT can be used as exogenously as endogenously to report benefits to the meat and meat products.

## 6. New Trends

New trends in the use of HXT in meat focus on the maintenance of its antioxidant power and eliminating its intense flavour and smell, as shown in the studies of Cofrades et al. [54] or Freire et al. [59]. At first, it was proved that HXT added to the ground pork meat in W_1_/O/W_2_ emulsions reduced lipid oxidation by more than 50%. In the same way, Freire et al. showed that using cold-set gelled double emulsions enriched with perilla oil (*Perilla frutescens* as a source of n-3 fatty acids) and HXT as animal fat replacer during 30 days of storage, it was found HXT maintained the stability of emulsions and increased its antioxidant capacity while it was reduced lipid oxidation and bacterial growth. That kind of emulsion has the characteristic of encapsulating the extract to avoid the strong flavour and odour of HXT on meat. However, there are no studies about sensorial changes after the application of the HXT extracts.

On the other hand, Moudache et al. [60] have studied another application of HXT: an antioxidant food packaging material applied to fresh minced pork and whose plastic film contained olive leave extract. This researcher showed that active films with olive leave extract, rich in HXT, had a positive effect on the oxidation stability of meat fat. In addition, this active packaging does not need being in contact with meat, so it does not alter the sensorial quality of the product.

## 7. Conclusions and Perspectives

As reviewed, the beneficial effects of HXT has been extensively investigated during the last twenty years, so many researchers of pharmaceutical industry have been focused on use of this compound due to its nutraceutical power. In parallel, researchers in the food and meat industries have been focused on the elimination of preservatives and dyes in order to achieve the ‘clean label’. However, HXT has a strong flavour so cannot be added directly to the meat. For this reason, researchers have currently been focused on the encapsulation of this extract and the production of emulsion gels to prevent the sensorial alteration of meat products. However, nothing has been concluded in this field about the organoleptic quality of the product. Therefore, it can be concluded that the best way to obtain its benefits on meat is endogenously, through the animal diet, or through its application in new packaging systems. 

## Figures and Tables

**Table 1:** Results on the applicability of HXT on meat and its effect on product quality.

Extract Form	Concentration (ppm)	Meat Product	Test Setup	Tested Parameters	Results	Reference
Commercial	10,000 30,000	Ground beef patties	Ground beef was mixed with extract and inoculated with *E. coli* (10^7^ cfu/g). Uniform patties were formed, cooked and shock-cooled.	Total viable count	It was enhanced cell count reduction (3% survivors detectable)	[53]
				Amine quantification	Reduction of amine formation (50.6%)	
Commercial	100	Pork meat with W_1_/O/W_2_ emulsions	Ground pork meat and fat were mixed with W_1_/O/W_2_ emulsions and chia oil. Emulsions were vacuum packaged and keep in chilled storage (4 °C) until analyses on the 1st, 7th, 19th, 28th and 39th days.	Light microscopy	Particle size in samples with HXT was higher (*p* < 0.05)	[54]
				Antioxidant activity: DPPH	Chia oil presence in meat samples increased oxidation, however, HXT acted as antioxidant (8%).	
				Lipid oxidation (TBARs)	HXT presence in meat samples reduced lipid oxidation by more than 50%.	
Concentrate	100.23	Fermented sausages	During the drying process fermented sausages were dipped in extract solutions (2.5–5%) for 1 min at 20 °C and were continued drying.	Total viable count	No differences	[55]
				Lactic acid bacteria	No differences	
				Micrococci	Growth reduction affected volatile compound profile	
				Yeast	No significant differences	
				Moulds	Reduction of species	
				pH	No significant differences	
				Water activity	No significant differences	
				Lipid oxidation (TBARs)	Reduced values (12–38%)	
				Volatile compounds	Reduces volatile compounds from microbial esterification and lipid oxidation	
				Sensory attributes (colour)	Redness increased	
Commercial	100, 200, 400	Lamb meat patties	Minced lamb meat enriched in omega-3 fatty acids (with fish oil) was mixed with natural extracts and stored in high-oxygen modified atmosphere packs for up to 9 days at 4 °C.	In vitro antioxidant activity (ORAC and FRAP)	ORAC: No significant differences	[56]
					FRAP: antioxidant activity increases with extract presence	
				Colour (CIELab)	Lightness (L*) increased in samples without extract by changes in muscle proteins.	
					Significant differences between samples at day 3, 6 and 9 of storage.	
				Lipid oxidation (TBARs)	No significant differences	
				Protein oxidation (thiol and carbonyl groups)	Natural extracts improvement texture but it alteration odour and flavour.	
				Sensory analysis		
Concentrated, undefined	75, 150	Pork sausages	Ground pork (50/50 - meat/fat) was minced and mixed with salt and phenols. Mix was stuffed into 40-mm diameter bovine casings, were left to drip at 15 °C for 6 h and stored without packaging alternating fluorescent light (12 h dark and 12 h light) at 2 °C for 14 days. After, sausages were cooked, stored 72 h at 4 °C and frozen until analysis (80 °C)	Nutritional composition	No differences	[57]
				pH	No differences	
				Cooking loss	No differences	
				Diacylglycerols	Phenols had an inhibitory effect on microorganisms and a reduction in lipolytic activity.	
				Lipid oxidation (TBARs)	Oxidation was reduced (>40%) as TBARs as POV. But there are no differences in COPs.	
				Peroxide value (POV)		
				Cholesterol oxidation products (COPs)		
				Sensory analysis	Phenols presence was valorated worst by panellists.	
Commercial 1. HXT 23% from olive waters 2. HXT 7% from olive leaves 3. HXT 7% from olive waters	50	Chicken sausages	Pork fat and chicken meat were minced and mixed with walnuts, EVOO and three HXT extracts. Samples were cooked for 3 h at 72 °C, packaged in MAP (10% O_2_/20% CO_2_/10% N_2_) and stored at 4 °C for 21 days.	Nutritional composition	No differences	[21]
				Colour (CIELab)	L* and b* were lower in samples with HXT and EVOO, while a* was higher	
				Cooking loss	Cooking loss values were higher in samples with HXT	
				Lipid oxidation (TBARs)	In samples with HXT TBARs value was lower and in samples with HXT and EVOO, lipid oxidation was 71% lower than control.	
				Protein oxidation (thiol groups)	HXT reduced protein oxidation between 13–25%.	
				Scanning electron microscopy	Sausages incorporated HXT showed different structures.	
				Sensory analysis	Samples with HXT 7% from olive water was accepted, while other samples with HXT presented lowest acceptability.	
Commercial, 7% from olive waters	50	Chicken Frankfurters	Back fat and chicken meat were minced and mixed with walnuts, EVOO and HXT. Samples were cooked for 3 h at 72 °C, packaged in MAP (10% O_2_/20% CO_2_/10% N_2_) and stored at 4 °C for 21 days.	Nutritional composition	No differences	[20]
				Mineral content	Ca, K, Fe, Mg, P, Mn and Zn concentrations were higher in samples with HXT.	
					No differences	
				Fatty acid profile. Sensory analysis	Extracted flavour and odour parameters were increased in samples with HXT but it was accepted by panellists.	
Olive cake applied in chicken feed	4.6 9.5	Chicken meat	297 chickens were feeding until 21 days of age with three treatments: basal diet, diet supplemented with 82.5 g/kg olive phenols and diet supplemented with 165 g/kg olive phenols. Chickens were weighed at 28, 35 and 42 days of age and slaughtered at 42th. Carcasses were maintained at −20 °C for three months until consumption and at −80 °C for other analyses.	Chicken weight	The chicken weight was higher	[58]
				Colour (CIELab)	L* and b* was higher while a* was lower	
				Cooking loss	No differences	
				Nutritional composition	No differences	
				pH	No differences	
				Lipid oxidation (TBARs)	Lipid oxidation was lower	
				Antioxidant capacity (DPPH)	Samples with HXT showed a high antioxidant capacity	
				Sensory analysis	No differences, so HXT did not alter the sensory quality.	
